# Is psychosexual therapy a reliable alternative to bupropion extended-release to promote the sexual function in infertile women? An RCT

**DOI:** 10.18502/ijrm.v18i3.6714

**Published:** 2020-03-29

**Authors:** Hajar Pasha, Zahra Basirat, Mahbobeh Faramarzi, Farzan Kheirkhah

**Affiliations:** ^1^Infertility and Reproductive Health Research Center, Health Research Institute, Babol University of Medical Sciences, Babol, Iran.; ^2^Social Determinants of Health Research Center, Health Research Institute, Babol University of Medical Sciences, Babol, Iran.; ^3^Departments of Psychiatry, School of Medicine, Babol University of Medical Sciences, Babol, Iran.

**Keywords:** Infertility, Sexual activities, Drug therapy, Psychotherapy, Bupropion.

## Abstract

**Background:**

Various treatment methods are used to deal with sexual problems.

**Objective:**

This study was applied to answer the question of whether psychosexual therapy (PST) can be a reliable alternative to bupropion extended-release (BUP ER) to promote sexual function in infertile women.

**Materials and Methods:**

In this randomized clinical trial, 105 infertile women with sexual dysfunction were randomly allocated to three groups: PST, BUP ER, and a control group. The PST group participated in a total of eight 2-hr group sessions. In BUP ER group, 150 mg/day Bupropion ER was administered for eight weeks. The control group did not receive any interventions. The female sexual function index (FSFI) and a clinical interview were used to assess their sexual dysfunction.

**Results:**

The mean pre-to-post treatment scores of FSFI and its subscales increased significantly in PST and BUP ER groups (except in the subscale of sexual pain) (p = 0.0001, p = 0.0001). The changes in the subjects were not significant in the control group. After adjusting for the baseline values, the results remained significant for the mean FSFI (p = 0.0001), and its subscales between the groups. Compared to the control group, a significant increase was observed in the mean FSFI (p = 0.0001, p = 0.002) and its subscales in the PST group and in the BUP ER group (except in the subscale of sexual pain). Comparison of two intervention methods showed that PST had the better effect on the sexual function improving (p = 0.0001) and its subscales (exempting the subscale of orgasm) than BUP ER.

**Conclusion:**

PST can be considered not only a reliable alternative to pharmacotherapy; it also produces better results in terms of improving sexual function in infertile women.

## 1. Introduction

Sexuality is an important part of health and the main subject in quality of life studies (1). Sexual health is defined as the “ability to have enjoyable and safe sex life” (2). The cycle of a woman's sexual response is mainly affected by psychological, social, and physical factors (3). Female sexual dysfunction (SD) is a common health problem (4) that includes female sexual interest/arousal disorder, female orgasmic disorder, and genito-pelvic pain/penetration disorder (5). Reviews of the literature showed that approximately 30–50% of women have sexual complaints (1). SDs are particularly common among infertile women. A recent study reported the prevalence of SDs as 46.6% in a sample of healthy Iranian infertile women. Infertility is a stressful and difficult phenomenon and may also become a source of SD (4), although, it is equally probable that sexual problems directly affect infertility by decreasing the intercourse frequency (6). SD is a widespread health problem calling for effective treatment options (4, 7). Studies reveal that both psychotherapy and pharmacotherapy can improve sexual problems. The sex therapy approach of Masters and Johnson was the first psychological intervention (encompassing counseling, psychosexual education, behavioral exercises, and sensate focus) that facilitated the re-experience of sexual pleasure (8). Franken proposed that sexual interventions can promote safe sex, give rise to sexual identity, and prevent SD (9). Studies have provided supporting pieces of evidence on the benefits of bupropion for SD (10, 11). This medication has been proven effective in the treatment of the clinical signs of female SD (11, 12) and has improved sexual functioning in 29% of non-depressed women with hypoactive sexual desire disorder within a matter of a few weeks (13). Furthermore, a study has shown the benefit of bupropion as an antidepressant in women who are depressed or who have selective serotonin reuptake inhibitors -induced SD (10). Primarily, bupropion is a dopamine agonist and raises the dopamine level, which is a key mediator of several human behaviors, including sexual function (11).

To our knowledge, there are no randomized, controlled, prospective trials published on the effectiveness of psychosexual therapy (PST) in comparison with pharmacotherapy in improving sexual function in cases of infertility. The present study was therefore conducted as a randomized clinical controlled trial to examine the effectiveness of PST and pharmacotherapy with bupropion extended-release (BUP ER) in promoting sexual function in infertile women. The study hypothesizes that PST is a reliable alternative to BUP ER for promoting sexual function in infertile women.

## 2. Materials and Methods 

An open-label randomized, controlled, clinical trial was conducted in the Infertility and Reproductive Health Center of Babol University of Medical Sciences, Babol, Iran, from December 2014 to July 2015. The study is focused on the comparing of pharmaceutical and non-pharmaceutical methods in promoting sexual function in infertile women. Two midwives with no clinical involvement in the trial enrolled the participants and assigned them to the interventions. Our inclusion criteria included the female sexual function index (FSFI) ≤ 26.55 (Final approval by a female psychologist in the clinical interview), the infertility duration more than one year, no intention of infertility treatment over the next two months, age < 45 yr., reading and writing literacy, having a stable sexual life (At least for four weeks before study), no history of remarriage in husband, no history of sterilization, and having no foster children. The infertile women with any of the following conditions were excluded: history of hypertension, diabetes mellitus, cardiovascular disease, hypothyroidism, hyperthyroidism, epilepsy, spinal cord injury, liver dysfunction, psychiatric problems under treatment, having suicidal ideation, taking medications that cause SD such as barbiturates, benzodiazepines, antidepressants and antihypertensive drugs, getting psychological support (such as psychotherapy sessions, relaxation exercises, Yoga, and etc.), having a stressful event in the past three months (serious illness or death in the family), major changes in living conditions, and smoking.

In addition, all participants with Beck depression score ≥ 10, who met the criteria for major depression in the clinical interview by a female psychologist, were excluded from the study and were referred to a psychiatrist for treatment.

**Figure 1:**
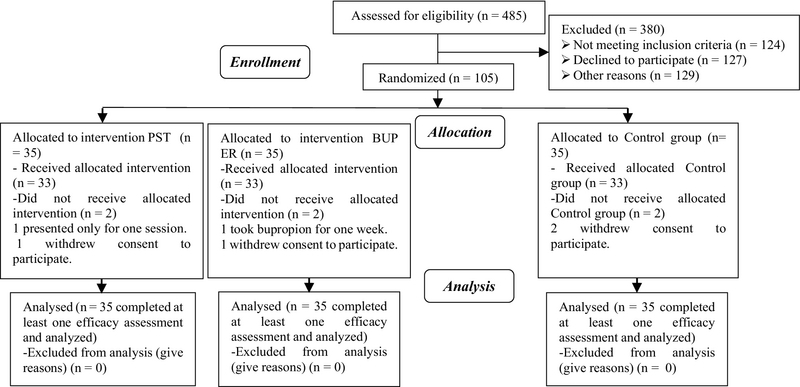
The recruitment and randomization of the infertile women in the study group.

Of the 485 infertile women referred to the Infertility and Reproductive Health Center from December 2014 to July 2015, 127 were not willing to participate, 124 met the exclusion criteria, and 129 had no SD. A total of 105 women with SD agreed to participate in the study and only 99 participants remained until the end of the study (Figure 1). In this protocol, both the researcher and the participants were not blinded. The sample size was calculated as 23 subjects in each group to yield an 80% power with 95% Confidence level, accuracy = 6.02, and approximate standard deviation (SD) = 7.3, based on previous studies for each group (8, 10, 12-13). Considering the drop out percentage as 10 % and using the corrected sample size formula (n' = Kn, k = 2) (14), a total of 105 eligible infertile women were selected equally in three groups (n = 35/each group).

Computer-aid randomization procedures (by an investigator with no clinical involvement in the trial) were used at the beginning of the treatment phase to allocate the participants in a 1:1:1 ratio into the three groups of 35 people each – a PST group, a BUP ER therapy group, and a control group. The PST group participated in a total of eight 2-hr group sessions of mindfulness-based cognitive therapy (MBCT), behavior sex therapy, and relaxation training presented in the form of lectures, question and answer, group discussions, booklets, and CD. There were 9 to 13 members in each session. The therapeutic model used was a combination of MBCT (15) and behavior sex therapy. The sexual behavior program and the relaxation training sessions were adapted for infertile women based on the Crowe and Ridley model and also tranquility books (16, 17).

In BUP ER group, 150 mg/day Bupropion ER (Wellban External Release, Dr. Abidi Pharmaceutical Laboratories, Iran) was administered under the supervision of a psychiatrist for eight weeks. In previous studies, the trial length varied from 4 to 24 weeks with bupropion dosage ranging from 75 to 450 mg/day (10, 12-13, 18, 19). The control group did not receive any interventions. To observe the ethics of research, the members of the control group were referred to a sex therapy clinic at the end of the eight-week trial and educational package was given to them. Regular monitoring of adverse events, height, body weight, and vital signs were assessed in each groups by the researcher once every two weeks. The participants were informed not to use any other drugs. To answer any further questions, the researchers gave their phone numbers to the participants. Also, the researchers monitored participants for any change in their health status or for any complaint by phone weekly.

A demographic questionnaire was used to collect participants' socio-demographic data, including age, husband's age, level of education, and level education of husband, husband's occupation, socioeconomic status, duration of infertility, and the type of infertility. FSFI questionnaire was also used to collect data on the participants; this self-report inventory consists of 19 items and was developed by Rosen and his colleagues (20). This study used the culturally adapted Iranian Version of the FSFI (IV-FSFI). This inventory has six different subscales, including desire, arousal, lubrication, orgasm, satisfaction, and pain. The responses to the items are scored based on a 5-point Likert-type scale. The total score obtained ranges from 2 to 36. The participants scoring 26.55 or less are considered “at risk” for SD. A higher FSFI score indicates a better sexual function. The reliability of the IV-FSFI has been confirmed with a Cronbach alpha ranging from 0.79 to 0.86 and the tool has excellent construct validity as well (4, 20). The BDI is used to assess the severity of depression symptoms with reliability (0.96), validity (0.89) in the Iranian population (21).

### Ethical consideration

The research project has been approved by the Ethics Committee of the Babol University of Medical Sciences, Babol, Iran (code: 4930). All participants were briefed on the study objectives and methods and were then evaluated to see whether they met the study inclusion criteria. Informed written consent forms were obtained from all the participants. Data were collected confidentially.

### Statistical analysis

The Chi-squared test, the paired *t* test, the Analysis of covariance (ANCOVA), the Multivariate Analysis of Covariance (MANCOVA), and post-hoc test were used for analyzing the data. Demographic variables were analyzed using descriptive statistics (i.e., mean and standard deviation for the quantitative measurements and percentage for the categorical data). Before-and-after means comparisons in each group were made using the paired sample *t* test. The mean difference in the sexual function score was compared using the ANCOVA, which included region and baseline value as the covariate. The MANCOVA was used if the correlation between the FSFI subscales (desire, arousal, lubrication, orgasm, satisfaction, and pain) ranged from 0.3 to 0.7. The statistical procedure assumed randomized sampling, the normality of distributions, the homogeneity of variances as per Levene's F test, and linear relationship between the dependent variable and the covariate. Post-hoc test was used to calculate pairwise comparisons between groups. The skewness and kurtosis test was used to evaluate the normality of the continuous variables. The study population in which data were analyzed consisted of the intent-to-treat (ITT) population, that is, of all the patients randomized to the treatment groups. There were no differences in the demographic details and the baseline FSFI and subscale scores in the subjects who stayed in the study until the end and those who did not. All the analyses were performed using the Statistical Package for the Social Sciences, version 21.0 (SPSS, IBM Crop., Armonk, NY, USA). P < 0.05 was considered as significant.

## 3. Results

The mean age (±SD) of the subjects and their husbands was 28.89 ± 5.42 and 32.05 ± 5.14 yr., respectively. The majority of the infertile women and their husbands had a high school diploma (50.5% and 35.25, respectively). More husbands had university education than wives (30.5% vs. 26.7%, respectively; p < 0.001).

The mean duration of infertility was 4.39 ± 3.54 yr (ranging from 2 to 16 yr) in the subjects. There were no significant differences between the baseline FSFI and the socio-demographic data of the three groups in terms of age, husband's age, level of education, husband's level of education, and the duration of infertility (Table I)

The results of the present study showed significant differences in the mean of FSFI score between the groups after adjusting for baseline values in the ANCOVA (p < 0.0001). The research assumption about the effect of the therapeutic intervention on FSFI with a probability value above 99% in infertile women was thus accepted. The pair-wise ANCOVA comparison of the post-intervention FSFI scores showed a significant difference in PST and BUP ER groups compared to the control group (p < 0.0001; p = 0.002, respectively) and also between the treatment groups themselves as per the post-hoc ANCOVA (p < 0.0001; Table II). A significant difference was observed in the mean FSFI subscale scores between the groups after adjusting for baseline values in the MANCOVA (desire, excitement, lubrication, orgasm, satisfaction, p < 00001, sexual pain; p = 0.002). Table III presents the adjusted mean (±SD) and MANCOVA of the dependent variables (desire, excitement, lubrication, orgasm, satisfaction, and pain). The pair-wise MANCOVA comparison of the post-intervention FSFI subscale scores showed significant differences in the PST and BUP groups (except in the subscale of pain) compared to the control group as well as between the treatment groups themselves (except in the subscale of orgasm).

The mean (±SD) of the FSFI score was 22.86 ± 2.29 in infertile women. The mean and standard deviation of the FSFI score was 22.56 ± 2.36 at baseline and 27.73 ± 3.78 after the intervention in treatment groups (PST, BUP ER; p < 0.0001) (Table II). The paired *t* test showed significant changes in mean pre-to-post treatment scores of FSFI (p < 0.0001) and the scores obtained in the subscales of desire, arousal, lubrication, orgasm, and satisfaction in both the PST and the BUP groups (p < 0.0001). The changes in the subjects were not significant in the control group. A comparison of the mean baseline and post-intervention scores of the subscale of pain using the paired *t* test showed a decreased pain in the PST group (p < 0.0001). The changes in the subjects were not significant in the BUP (p = 0.315) and control (p = 0.245) groups. BUP ER was well tolerated.

**Table 1:** The sociodemographic characteristics of the study participants


**Variable**	**All (n = 105)**	**Treatment groups (n = 35/each)**			
		**PST**	**BUP ER**	**Control**	**P-value**
Age (yr)*	28.89 ± 5.42	30.00 ± 5.23	27.31 ± 5.22	29.34 ± 5.58	0.096
Age of husband (yr)*	32.05 ± 5.14	33.11 ± 5.68	30.60 ± 3.59	32.43 ± 5.68	0.106
Infertility duration (yr)*	4.39 ± 3.54	4.11 ± 3.55	4.97 ± 3.39	4.09 ± 3.68	0.497
Educational level**					
**< Diploma**	24 (22.9)	10 (28.6)	10 (28.6)	4 (11.4)	
**Diploma**	53 (50.5)	17 (48.6)	18 (51.4)	18 (51.4)	
**College education**	28 (26.7)	8 (22.9)	7 (20.2)	13 (37.1)	0.262
Husband educational level**					
**< Diploma**	36 (34.3)	12 (34.3)	12 (34.3)	12 (34.3)		
**Diploma**	37 (35.2)	15 (42.9)	12 (34.3)	10 (28.6)	
**College education**	32 (30.5)	8 (22.9)	11 (31.4)	13 (37.1)	0.696
*Data presented as Mean ± SD. ANOVA was performed to compare the mean scores of groups **Data presented as n (%). A Chi*-*square test was performed to compare the frequencies of groups PST: Psychosexual therapy; BUP ER: Bupropion extended-release					

**Table 2:** Adjusted mean (SD) and analysis of covariance of FSFI in the groups


**Variable**	**Treatment groups (n = 35/each)**						**Sum of squares**	**Mean square**	**df**	**F statistics**	**Observed power**	**P-value**
	**PST**		**BUP ER**		**Control**				
	**Pre**	**Post**	**Pre**	**Post**	**Pre**	**Post**			
**FSFI**	22.20 ± 2.54	29.10 ± 3.48	22.92 ± 2.13	26.35 ± 3.60	23.46 ± 2.06	24.18 ± 3.19	530.565	265.283	2	25.982	0.999	0.0001
	**Pair wise comparisons**											
**Group**	**PST vs control**				**PST vs BUP ER**				**BUP ER vs Control**			
	**P-value**				**P-value**				**P-value**			
**FSFI**	0.0001				0.0001				0.002			
FSFI: Female sexual function; PST: Psychosexual therapy; BUP ER: Bupropion extended-release Note. Comparing the change in the pre- and post-mean FSFI score, the difference was significant in each treatment groups (PST; p < 0.0001 and BUP; p < 0.0001), but not in the control group (p = 0.185) (Paired *t* test)												

**Table 3:** Adjusted mean (SD) and multivariate analysis of covariance of FSFI domains in the groups


**Variable**		**Treatment groups (n = 35/each)**						**Sum of squares**	**Mean square**	**df**	**F statistics**	**Observed power**	**P-value**
	**PST**		**BUP ER**		**Control**				
	**Pre**	**Post**	**Pre**	**Post**	**Pre**	**Post**			
**Subscales**													
	**Desire**	3.14 ± 74	4.20 ± 0.91	3.34 ± 0.65	3.89 ± 0.76	3.14 ± 0.75	3.38 ± 0.51	11.784	5.892	2	11.156	0.991	0.0001
	**Excitement**	3.3 ± 0.76	4.41 ± 0.88	3.19 ± 0.63	3.89 ± 0.82	3.10 ± 0.59	3.33 ± 0.72	19.240	9.620	2	15.412	0.999	0.0001
	**Lubrication**	4.12 ± 0.82	5.08 ± 0.64	4.21 ± 0.78	4.71 ± 0.86	4.32 ± 0.70	4.40 ± 0.73	9.230	4.615	2	9.814	0.981	0.0001
	**Orgasm**	3.79 ± 1.05	4.98 ± 0.84	3.79 ± 0.89	4.62 ± 0.75	4.14 ± 0.77	4.14 ± 0.89	13.417	6.709	2	10.444	0.986	0.0001
	**Satisfaction**	3.8 ± 0.91	5.17 ± 0.83	4.04 ± 0.93	4.67 ± 0.79	4.35 ± 0.79	4.33 ± 0.97	12.697	6.349	2	9.074	0.971	0.0001
	**Pain**	3.92 ± 1.46	5.26 ± 1.09	4.34 ± 1.39	4.57 ± 1.11	4.40 ± 1.17	4.59 ± 1.29	13.249	6.625	2	6.567	0.902	0.002
**Pair-wise comparisons**													
**Subscales**		**PST vs Control**				**PST vs BUP ER**				**BUP ER vs control**			
	**P-value**				**P-value**				**P-value**			
	**Desire**	0.0001				0.028				0.009			
	**Excitement**	0.0001				0.007				0.004			
	**Lubrication**	0.0001				0.019				0.031			
	**Orgasm**	0.0001				0.070				0.005			
	**Satisfaction**	0.0001				0.024				0.037			
	**Pain**	0.003				0.002				0.938			
PST: Psychosexual therapy; BUP ER: Bupropion extended-release Note. The paired *t* test showed significant changes in the mean pre-to-post treatment scores obtained in the subscales of FSFI in the PST and BUP ER (except in the subscale of sexual pain) (p < 0.0001), but not in the control group													

## 4. Discussion

The results of the present study revealed a significant difference in the post-intervention score of FSFI and its subscales (desire, arousal, lubrication, orgasm, satisfaction, and pain) between the three groups after adjusting for baseline values. The increasing scores were more significant in the treatment groups compared to the control group. SD is prevalent in infertile women and calls for the design of effective treatment options (7). Female sexuality can be affected by psychological and physical factors (3); therefore; psychotherapy and medical treatment can both be suitable choices for reducing sexual concerns (8). Psychotherapy, targeted sexual therapy, and pharmacologic treatments are the currently available interventions for the management of SD in women (22). Studies have revealed pharmacological and non-pharmacological interventions such as sex education programs, cognitive behavioral therapy (CBT), and chemical pharmacotherapy to be associated with greater marital satisfaction and improved sex life (19, 23).

The present study found that eight weeks of PST yields statistically and clinically significant improvements in sexual function compared to when no interventions are performed. The analysis of the pre-to-post interventions data showed significant changes in sexual function scores in the PST group, whereas this change was not significant in the control group. Fruhauf and her colleagues found that psychosexual interventions can help promote sexual function and as such constitute a promising treatment option (8). Psychotherapy or sexual therapy is beneficial for SD in women and sex therapy appears to be an effective treatment option for improving sex life (24). PST is therefore helpful and necessary, especially for infertile women.

According to the findings, PST had a significant effect on all the subscales of desire, excitement, lubricant, orgasm, satisfaction, and pain compared to the control group. A comparison of the mean baseline and post-intervention subscale scores of FSFI in the PST group showed an increasing trend. There were no significant changes in the FSFI subscale scores in the control group. A review of the literature showed that psychological interventions increase sexual desire, orgasm, and sexual satisfaction and decline the symptoms of SD (8, 24). In a review study, Fuql-Meyer and colleagues recommended the comprehensive somato-psychological multidisciplinary approach for the management of female genital sexual pain (25). Sex education programs significantly improved the overall score of sexual function as well as the scores of all its subscales, including desire, excitement, lubrication, and satisfaction (23). Mohammadi and colleagues showed that sexual skills training helps increase women's marital satisfaction (26). In a trial study conducted by Eshghi and colleagues, sexual CBT for couples helped to improve hypoactive sexual desire disorder in women (27), while in another study, no significant changes were observed in the subjects in the subscales of orgasm and pain after receiving a sex education intervention (23).

The results of the present study showed a significant improvement in sexual function in the BUP ER group compared to the control group. The findings showed that the pre-to-post treatment sexual function score changed significantly in the subjects in the BUP ER group, whereas these changes were not significant in the control group. In line with these findings, Ginzburg and colleagues also reviewed studies published in Medline from 1970 to 2005 and found that bupropion has a favorable effect on SD (19). In a double-blind multicenter study, Thase and colleagues found that the total score of sexual function increases after a regimen of bupropion (28). A number of trial studies have also shown that bupropion may be a promising medication for the treatment of sexual problems (12, 19). Koshino and colleagues, however, reported no significant changes in the subjects treated with bupropion in regard to SD, which is inconsistent with the present findings (29). Bupropion is a dopamine agonist and raises dopamine levels. A dopaminergic pathway is essential to physiological mating behavior. A review of the literature found that pharmacological agents with central mechanisms of action or androgenic effects can be a good option for the treatment of different aspects of female SD (12). The effect of bupropion on sexual function is attributed to how it increases dopaminergic and noradrenergic transmission (11). Moreover, there was a significant improvement depression symptom. Lower depression score associated with higher sexual function. As bupropion is an antidepressant, it is therefore important to note that the promotion of sexual function may relate with a resolution of depression scores in a patient with SD (18), since depression and sexual problems are interchangeable with each other and treatment of one will change the others.

The obtained data revealed statistically significant improvements in sexual desire, arousal, lubrication, satisfaction, and orgasm (but not in pain) in the BUP ER group compared to the control group. In addition, a comparison of the mean baseline and post-intervention FSFI subscale scores showed an increase in all the subscales of FSFI (except in the subscale of pain) as the control group showed no significant changes in the mean FSFI subscale scores, which is nearly consistent with the results of previous studies on the subject (12, 13). A review of the literature showed that bupropion can be a promising medication for SD (19). An increase in sexual libido and arousal was reported in other studies following the use of this medication (10, 12, 18). Bupropion was also effective in treating hypoactive sexual desire disorder in women (10, 13, 30). Croft and colleagues, however, conducted a placebo-controlled trial and showed no statistically significant differences in sexual satisfaction between the placebo and bupropion groups after one week of treatment, and by the end of the study, the percentage of sexual satisfaction was similar in the placebo group and the bupropion group (18). Due to the lack of changes in the orgasm subscale, it can be concluded that reaching orgasm is a skill that requires practice, more time, and long-term medication, which may be beyond the short scope of this study and its short treatment periods. In one study, Pereira and colleagues found that diffuse long-term pain is more difficult to treat (30). Further studies need to detect factors contributing to the lack of an effect in the orgasm subscale.

According to the findings, the PST group showed superior results to the BUP ER group in terms of improved FSFI and subscale scores, exempting the subscale of orgasm. The mean total FSFI and subscale (desire, arousal, lubrication, satisfaction, and pain) scores were significantly higher in the PST group compared to the BUP ER group at the end of the study. A similar study showed that sexual and marital problems improve more extensively with CBT than with pharmacotherapy. CBT is, therefore, a reliable and superior alternative to pharmacotherapy in reducing sexual and marital concern in infertile women (31). Another study showed that psychological interventions are a promising treatment for sexual problems compared to pharmacological treatments and have two principal benefits, including a lack of physical side effects and the re-establishment of sexual function beyond a mere reduction of symptoms (7). Shams Mofaraheh and colleagues proposed marital counseling with sex education as one of the most effective treatment options for promoting sexual satisfaction and improving the quality of sex life (32). There is still no consensus on the effectiveness of pharmacotherapy and medications such as bupropion cost more than expected and are also associated with common adverse effects such as dry mouth, headache, nausea, and insomnia (12); as chemical options pose side effects, it is better to seek a second clinical opinion before administering them for the treatment of SD. Treatments for sexual problems can either include non-pharmacological or pharmacological measures. Among all the methods, cognitive behavioral measure has been recognized to be most useful (33), and PST can thus be regarded as a superior alternative to pharmacotherapy in the resolution of most aspects of female SD. Moreover, the present findings showed a trended significant difference in the scores of the subscale of orgasm in the PST group compared to the BUP ER group; this difference may be attributed to how PST requires a longer time to have its efficacy on orgasm confirmed. In a review of literature by Heiman and Meston, physical causes for primary orgasmic dysfunction were found to be rare and they can, therefore, be effectively treated through psychosexual interventions. The majority of the cases of intermittent or situational anorgasmia is attributed to relationship problems and can, therefore, be resolved with psychotherapy (33). In a randomized clinical trial study by Pereira and colleagues, sex therapy was found to lead to better outcomes for patients with orgasmic and sexual pain disorder (30). These findings support the benefits of PST compared to bupropion on female sexual function and its subscales in healthy infertile women.

##  Limitation

There were a number of limitations in this study protocol. The first limitation was the small sample size examined; although the select health center is the only public referral center for infertility and reproductive health in Babol, Iran; the results cannot be generalized to the entire population of infertile women with SD in Iran. Future studies are therefore recommended to consider the entire population of infertile Iranian women. The second limitation was the short period of the study; future studies need to examine the long-term sexual effects of psychosexual therapy and bupropion. The third limitation was that it is not clear whether or not the different results between the groups can be attributed to how more time and attention were given to the group of infertile women in the PST group. Moreover, the infertile women were not blinded to their method of intervention and there were also no placebo groups with which to compare the results. The researchers thus recommended double-blind, randomized, placebo-controlled, clinical trials to be conducted on the subject in the future. The strengths of this study include the use of a validated FSFI and a clinical interview.

## 5. Conclusion

To conclude, the results of the present controlled clinical trial showed that PST is superior to pharmacotherapy with BUP ER in improving sexual function. Pharmacotherapy with BUP ER is better than no therapy, although it does not affect pain during sex. This finding suggests that PST can be a good alternative for the treatment of infertile women with SD.

##  Conflict of Interest

The authors declare that there is no conflict of interest.
